# Infection Mitigation Efficacy of Photoactive Titania on Orthopedic Implant Materials

**DOI:** 10.4061/2011/571652

**Published:** 2011-03-10

**Authors:** Abdul-Majeed Azad, Ryan Hershey, Asem Aboelzahab, Vijay Goel

**Affiliations:** ^1^Department of Chemical Engineering, The University of Toledo, 2801 West Bancroft Street, Toledo, OH 43606-3390, USA; ^2^Vesuvius Research Center, 495 Emma Street, Bettsville, OH 44815, USA; ^3^Bioengineering Department, The University of Toledo, 2801 West Bancroft Street, Toledo, OH 43606-3390, USA; ^4^Departments of Bioengineering and Orthopedic Surgery, Health Science Campus, The University of Toledo, 2801 West Bancroft Street, Toledo, OH 43606-3390, USA

## Abstract

In order to impede infection and achieve accelerated wound healing in the postorthopaedic surgery patients, a simple and benign procedure for creating nanotubular or nanofibrillar structure of photoactive TiO_2_ on the surface of Ti plates and wires is described. The nanoscale TiO_2_ films on titanium were grown by hydrothermal processing in one case and by anodization in the presence of dilute mineral acids under mild and benign conditions in the other. Confocal microscopy results demonstrated at least 50% reduction in the population of *E. coli* colonies (concentration 2.15 × 10^7^ cells/mL) on TiO_2_-coated implants upon an IR exposure of up to 30 s; it required *∼*20 min of exposure to UV beam for the same effect. These findings suggest the probability of eliminating wound infection during and after orthopedic surgical procedures by brief illumination of photoactive titania films on the implants with an IR beam.

## 1. Introduction

Disease-carrying pathogens in the body not only destroy healthy tissue but can eventually multiply and spread throughout the blood stream causing infection. Infections can be reduced and healing accelerated by using a nanotechnological approach with photoactive antimicrobial materials.

We recently described the fabrication of pure and doped titania nanofibers possessing optimal porosity and structure, and their photocatalytic antimicrobial activity towards *E. coli* [1]. With the objectivity of their ultimate application towards mitigation of wound infection and bone healing—the spinal injury in particular—experimental protocols were developed to create photoactive films of titanium dioxide (titania) on titanium substrates. Several methods were developed for creating nanostructured coating of titania on “commercial purity” titanium (cp Ti) coupons and implants. The methods included hydrothermal processing under different experimental conditions using various media. In one case, titanium coupons precoated with TiO_2_ films were also used. As another viable technique, anodization was also employed to create nanotubular structures on titanium plates and wires. Exposure of these implants coated with titania films to IR laser for up to 30 s, demonstrated effective inhibition of *E. coli* growth, thus opening the possibility of using Ti implants coated with TiO_2_ nanocrystals or nanotubes as means of effective disinfectants for the prevention of major surgical site infections. The demonstration of the efficacy of IR light towards infection mitigation in terms of the biocidal activity of titania-coated implant material is the first of its kind.

## 2. Materials and Methods

Titanium—the basic material in implants—was used in two configurations: plates and wires. While plates are ideal for fixed geometry applications, it is anticipated that flexible wires would be a good way to induce healing in those orthopedic injuries where plates are difficult to be inserted. Wires can be folded, twisted, and configured to reach and stay in places where plates cannot. Another point of relevance that justifies using wires is the fact that the hip implants are coated with bead or wire geometry to provide surface for bone in-growth, thus, making antibacterial coatings on intricate yet flexible geometries such as wires valuable. With the same rationale, work is in progress using Ti mesh, and the results of this investigation would be reported elsewhere.

The desired coating on Ti substrates was prepared by using three methods: hydrothermal processing of cp Ti coupons (from Alfa-Aesar, MA, and United Titanium Inc., Wooster, OH, USA), hydrothermal processing of TiO_2_-coated cp Ti coupons (from Henkel Corporation, Madison Heights, MI, USA), and anodization of cp Ti coupons and cp Ti wires (Alfa-Aesar, MA, USA). In this study both plates and wires were 99.7% pure. One goal of the study was to optimize fabrication conditions which could be mimicked on the real-life Ti and Ti6AlV4 implants. Another goal was to examine which of the two morphologies (nanofibrillar growth via hydrothermal processing versus nanotubular growth via anodization) has higher bactericidal propensity. The coupons were cut from 100 mm × 100 mm × 2 mm Ti plates while the wire samples were 1.024 mm in diameter.

### 2.1. Hydrothermal Processing of cp Ti Plates

Hydrothermal processing is a homogeneous (for nanoparticles) or heterogeneous (for bulk materials) reaction in the presence of mineralizers under high-pressure and mild-temperature conditions in a closed system to dissolve and recrystallize materials that are slightly insoluble under normal conditions. Invariably, autoclaves are used for this purpose with suitable construction materials that could withstand temperatures up to ~250–300°C and pressures up to 2000 psi. The technique offers the advantage of upscaled production without much difficulty or compromising the consistency and reproducibility.

In our case, the purpose of hydrothermal synthesis was to create titania films on the Ti substrates. It was carried out in a bench-top 1-L capacity autoclave (Autoclave Engineers, Erie, PA, USA). The 2-mm-thick cp Ti plate was cut into about 6 mm × 6 mm coupons and polished unidirectionally with 400 grid SiC abrasive papers. This helped in creating some surface defects which are likely to assist in nucleation and growth of fibers during autoclaving. Coupons were rinsed in deionized (DI) water and dried in air. A small bucket-shaped reaction vessel designed and fabricated in the laboratory was used to conduct the hydrothermal reactions. The Ti coupons were placed in a bucket containing 20 mL of hydrogen peroxide (30% aqueous H_2_O_2_, Fisher Scientific, Waltham, MA, USA). The bucket was placed inside the main stainless steel autoclave, and the entire assembly was sealed. The reaction was carried out at 80°C (ramp rate of 3°/min) for 1 h. After the reaction was complete, the coupons were rinsed in DI water under sonication, dried in air, and heated in a split tube furnace (Lindberg Minimite, Asheville, NC, USA) at 700°C for 1 h with a ramp rate of 1°/min.

### 2.2. Hydrothermal Processing of TiO_2_-Coated Ti Plates

The Ti plates from Henkel Corporation (referred to as “Henkel plates” hereafter) had a thin film of TiO_2_ deposited on them by a patented aqueous plasma electrodeposition (PED) process in which the Ti substrate is made the anode and titanium-bearing compounds in solution are deposited and cured *in situ* by the plasma glow at the surface [2]. In a typical PED process, a pulsed DC voltage (240 V) was applied for 10 ms on and 30 ms off at a current density of approximately 1500 A/m^2^. This results in very adherent titania coatings on the cp Ti substrates. The as-received Henkel plates were cut into 6 mm × 6 mm pieces and cleaned in 0.1 M HCl (Fisher Scientific, Waltham, MA, USA, purity 37.5%) for 2 min followed by rinsing in DI water and acetone, respectively, and air drying. Prior to autoclaving, the Henkel coupons were heated at 800°C for 4 h in static air at a ramp rate of 10°/min. Retaining one for XRD and SEM analysis, the remaining coupons were chemically etched in 40% HF (Alfa-Aesar, MA, USA) solution for 10 s followed by rinsing in acetone and air drying. The heat-treated Henkel plates were subjected to autoclaving in 5 M NaOH (Fisher Scientific, Waltham, MA, USA) for 2 h at 100°C at a ramp rate of 3°/min. After the reaction was complete, the autoclaved coupons were washed and sonicated in acetone twice for 2 min.

### 2.3. Anodization of Ti Plates and Wires

**Figure 1:**
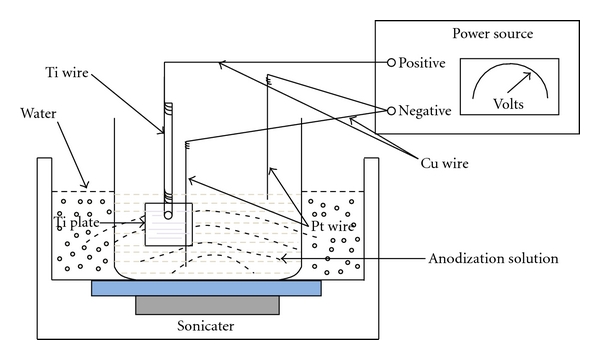
Schematic of the anodization setup.

Mohapatra et al. [3] have reported a method of creating TiO_2_ nanotubes on 0.2-mm-thick titanium foil by anodization in a mixture of 0.5 M H_3_PO_4_ and 0.14 M NaF solution at 20 V under sonication. In our work, we used a mixture of 0.5 M H_3_PO_4_ (Fisher Scientific, MA, USA) and 0.14 M HF (Alfa-Aesar, MA, USA) instead. Also, 2-mm-thick cp Ti plate instead of foil was used, and the voltage was maintained at 21 V. Sonication ensured that anodization took place evenly across the entire plate. In order to study the change in the quality of the oxide film formed on the surface, anodization was systematically carried out for 1, 2, and 4 h. Furthermore, one Pt wire on either side of the Ti plate was used to ensure that both sides were anodized. After each run, the anodized plate was thoroughly rinsed in DI water followed by chemical etching in a mixture of ethanol and 40% HF (5 mL each) for 5 s and washing again with DI water. Ti wires (1.024 mm in thickness) were also used in place of the Ti plate, in order to approximate the appropriate conditions required to produce the titania nanotubes. The Ti wires were anodized for 1, 2, and 4 h as well in a 0.5-M H_3_PO_4_ + 0.14 M HF mixture at a voltage of 21 V. An identical procedure was repeated with the actual implant specimen later.  Figure 1 shows the schematic of the setup used in this research. 

All the autoclaved and anodized samples were characterized by structural, microstructural, and quantitative analyses using X-ray diffraction (XRD- PANalytical X'Pert Pro MPD), Philips XL30FEG SEM or Hitachi S-4800 High-Resolution SEM, attached with the accessories capable of performing energy-dispersive spectroscopy (EDS) as well.

### 2.4. Preparation of the *Escherichia coli* (*E. coli*) Culture

The *Escherichia coli* #23724 bacteria were obtained in freeze-dried form (American Type Culture Collection, Manassas, VA, USA). The dry pellet was rehydrated in 2 mL of a super broth containing 32 g tryptone, 20 g yeast extract, and 5 g NaCl (all from Fisher Scientific, Waltham, MA, USA) per liter of aqueous solution made with ultra pure water with a conductivity of 5.5 × 10^−8^ S-cm. The super broth solution was sterilized in an autoclave at 121°C for 10 min at 18 psi, followed by cooling to room temperature (21°C) prior to storage in a refrigerator at 5°C.

The rehydrated *E. coli* was diluted with 100 mL of super broth and placed in a sterilized 250 mL flask. Sterilization was carried out by autoclaving the flask at 122°C for 20 min at 18 psi followed by drying for additional 20 min. The suspension was incubated in a shaker at 37°C for 12 h. For the restorage of the *E. coli*, the solution was spun down in a centrifuge until a pellet formed below a clear solution. The solution was decanted and pellet recovered, to which 10 mL of 10% glycerol (Fisher Scientific) solution of super broth was added to suspend the pellet again. The suspension was transferred to Eppendorf tubes and stored in a freezer at −80°C.

### 2.5. Bacterial Growth

For the bacterial growth, 100 mL of super broth was inoculated with a sterile 10 *μ*L loop (Fisher Scientific), followed by agitation at 150 rpm for 12 h until the bacteria culture reached the stationary phase. To determine the cell concentration, optical density at 600 nm was measured using a Perkin Elmer Multilabel Counter (Model Victor 3 1420) in solutions of various dilution of *E. coli*.

### 2.6. Staining of Bacteria

For the staining purposes, *E. coli* suspension containing 2.58 × 10^8^ cells/mL was used. It was diluted with 100 *μ*L of ultrapure water. Live/Dead BacLight Bacterial Viability Kits (Invitrogen, Carlsbad, CA, USA) provide a novel two-color fluorescence assay of bacterial viability that is useful for diverse bacterial genera. In this work, SYTO 9 and propidium iodide stains (1 *μ*L of each) were used. The *E. coli* was left in complete darkness for 15 min at 21°C to complete the staining.

In order to photoactivate the Ti plates and wires (against the *E. coli* suspension) onto which titania structure was created, an IR laser (*λ* = 808 nm; power = 1 W; www.freaklasers.com) was used. It should be pointed out that this IR laser is different from the IR flashlight used in our previous work [1] for activating the TiO_2_ nanofibers. The main reason for changing the source from IR flashlight (for fibers) to the IR laser (for films) is that the intensity of the beam from the flashlight was not strong enough to activate the TiO_2_ nanofibers or nanotubes formed on the latter. The focus beam size of the IR laser was adjusted to be about 5 mm diameter. The spatial confinement of the laser beam allowed the excitement of the titania plate alone while causing low or no direct phototoxicity to the bacteria.

For the purpose of evaluating the bactericidal efficacy of titania films formed on the Ti plate and/or wire, 40 *μ*L of the *E. coli* broth was pipetted in a petri dish. The IR laser was placed ~1 inch above the Ti coupon/wire and was turned on for 12 to 30 s. This allowed the titania film to be photoactivated. After irradiation, the plate was placed inside the petri dish containing the *E. coli* broth. The petri dish was then placed appropriately within the confocal microscope. The activity with respect to the bacterial colony was captured for the next several minutes (up to ~50 min). The reason for using the IR laser (*λ* = 808 nm) is owing to its effectiveness in the photoactivation of green fluorescent proteins [4]. After several iterations, it was found that excitation by the IR laser for 12 to 30 s was adequate for causing effective bacterial death. Since the majority of bactericidal experiments using titania photocatalyst to date have employed a UV source [5–9], one set of data was created by using a handheld UV light source (*λ* = 365 nm; Spectroline, model ENF-260C, Westbury, NY, USA) for ready comparison of the results. In the case of UV exposure, excitation duration was varied between 3 s and up to 20 min.

### 2.7. In-situ Image Analysis

In order to view the fluorescence given off by these stains, the dish containing titania plates/wires submerged in the *E. coli* suspension was positioned centrally in the multiphoton laser scanning confocal microscope (model Leica TCS SP5 MP, Leica Microsystems, Bannockburn, IL, USA). This instrument allows adapting the multiphoton system by optimally choosing from IR to picosecond or femtosecond laser and performing experiments with minimal phototoxicity. The reduced phototoxicity due to spatial confinement of excitation is ideal for living cells, which is crucial for this work in order to establish unequivocally that the observed bactericidal effect is due to the photocatalytic artifact of the titania nanofibers upon excitation and not due to photons alone. Upon photoexcitation, the *E. coli* stained with SYTO9 fluoresces green and that stained with propidium iodide fluoresces red. The excitation and emission of SYTO9 occurs at 480 and 500 nm, respectively. The excitation and emission of propidium iodide occurs at 490 and 635 nm, respectively.

The survival rate of the microorganism was determined by using the imaging software called ImageJ (image processing and analysis in Java—National Institute of Health, Bethesda, MD, USA). This software allows one to count the bacteria in order to determine the amount of live cells compared to total number of cells.

## 3. Results and Discussion

The titania films grown by various techniques described in the previous section on Ti coupons, wires, and on implants were characterized by multiple techniques, such as XRD, SEM, elemental mapping, and energy-dispersive spectroscopy (EDS). It should be pointed out that the identification of titania films grown on Ti substrates by hydrothermal processing and anodization, by XRD proved to be challenging; since the oxide coating in each of these cases was very thin and the X-rays penetrated past the TiO_2_ surface, thereby showing the diffraction pattern of titanium lying beneath. Hence, the XRD patterns are not shown. Collecting XRD patterns on wire samples was also challenging.

### 3.1. Microstructural Features of Hydrothermally Produced Titania Films Created on Ti Substrates

**Figure 2:**
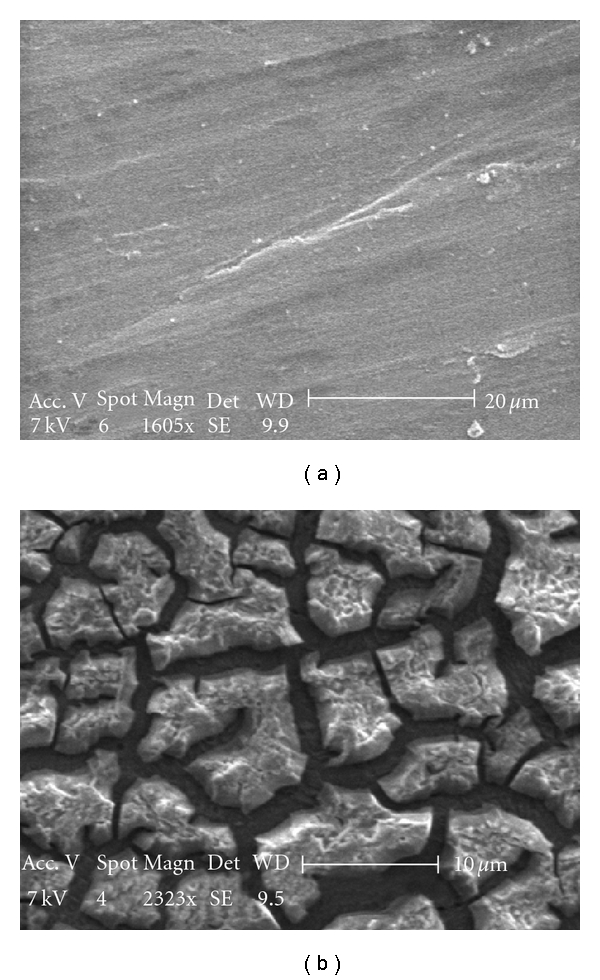
SEM images of the as-received (a) and autoclaved (b) Ti plates.

The SEM images of as-received and hydrothermally processed (in 30% H_2_O_2_ solution at 80°C for 1/2 h) Ti plate are shown in Figure 2. 

**Figure 3:**
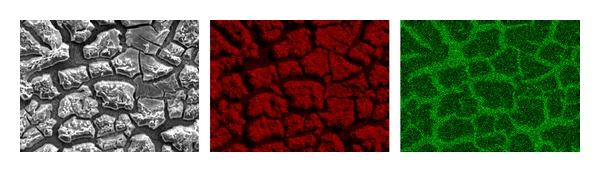
Elemental mapping of TiO_2_ coating on autoclaved Ti plate (O: red, Ti: green).

The elemental mapping in the hydrothermally processed plate with respect to titanium and oxygen shown in Figure 3 is indicative of the formation of oxide film.

**Figure 4:**
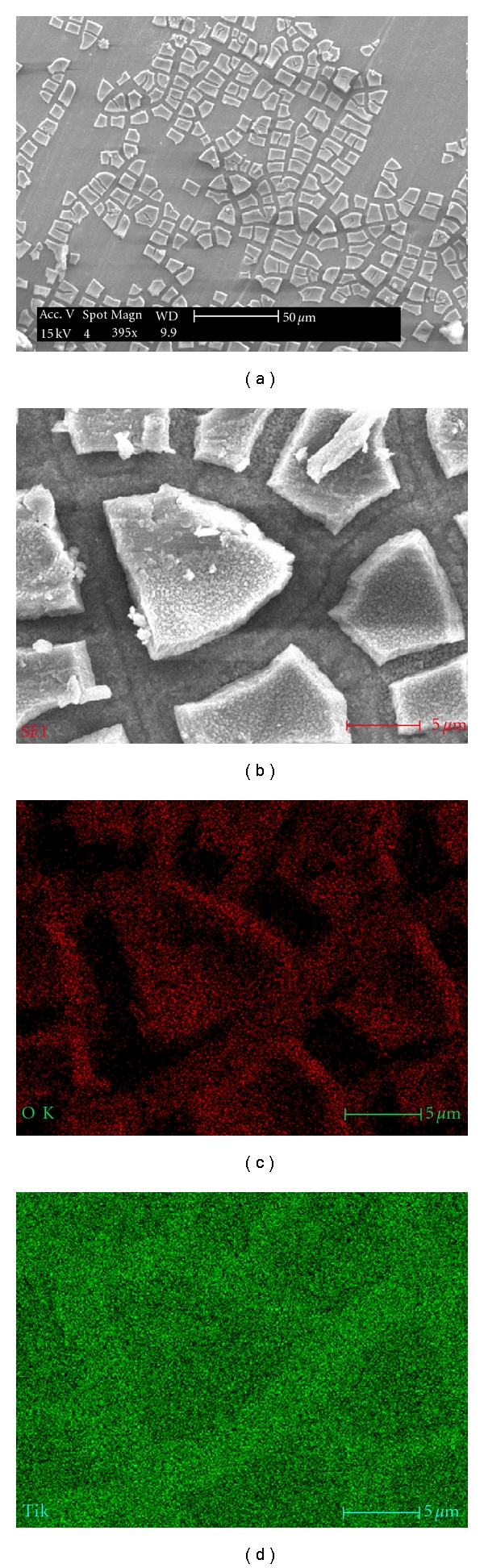
SEM images and elemental mapping of TiO_2_ film formed upon autoclaving in 30% H_2_O_2_ solution at 80°C/1 h (O: red, Ti: green).

Upon varying, the time for autoclaving is increased, the film morphology as well as the elemental concentration in the film also changed, as seen from Figure 4, for Ti coupons autoclaved in aqueous H_2_O_2_ solution at 80°C for 1 h. This is corroborated by the fraction of O and Ti in the two films, as shown in Table 1. 

Evidently, on hydrothermal processing for longer duration, greater oxidation of titanium is facilitated which leads to the increase in oxygen content of the film. The composition of the film obtained after 1 h of autoclaving at 80°C is closer to the stoichiometric titania; theoretically, a film of stoichiometric TiO_2_ contains 60 wt.% (33 at.%) of Ti and 40 wt.% (67 at.%) of O. However, the films formed in either case were found to be amorphous; to be photoactive, titania must be crystalline. Therefore, the Ti plates hydrothermally processed at 80°C for 1 h were calcined at 700°C for 1 h (ramp rate: 1°/min) after cleaning with DI water under sonication.

### 3.2. Hydrothermally Treated Henkel Plates

**Figure 5:**
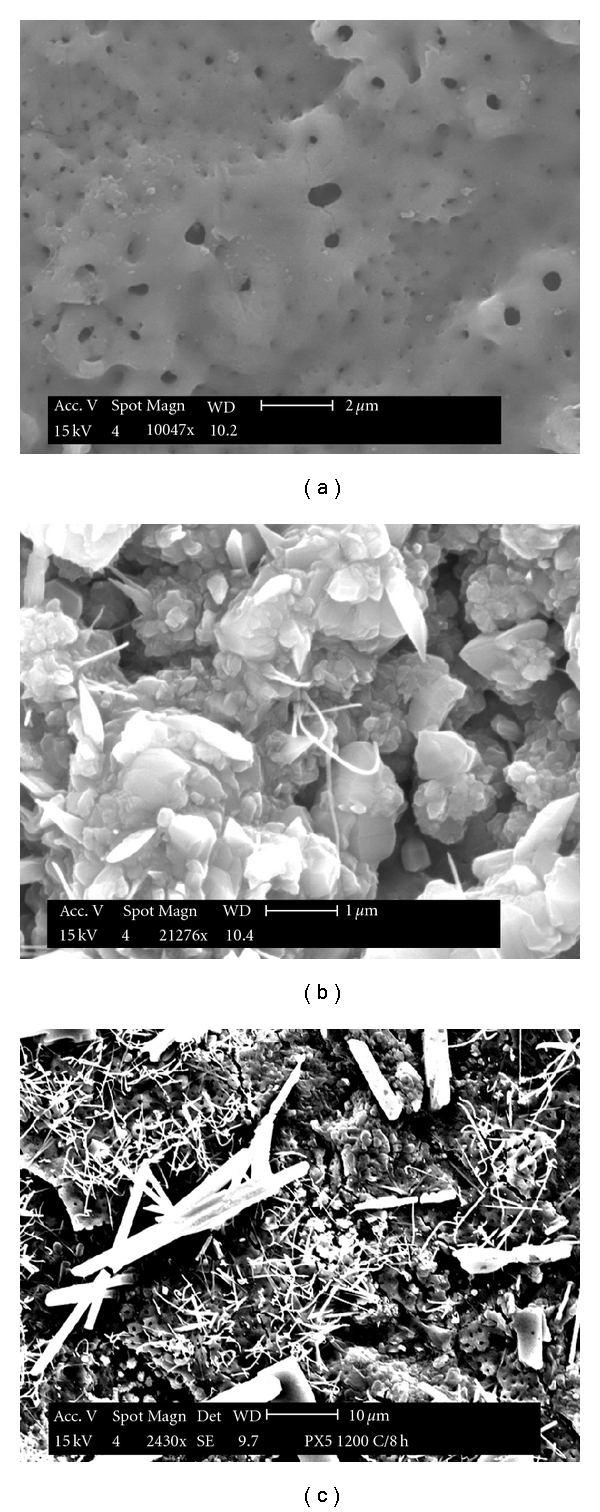
Morphological features in the: (a) as-received, (b) calcined, and (c) autoclaved Henkel plates.

As stated earlier, the Ti coupons from Henkel Corporation (Madison Heights, MI, USA) are coated with a thin film of TiO_2_ (structurally, brookite [2]). These were heated at 800°C for 4 h in static air at a ramp rate of 10°/min. A comparison of microstructural features between the as-received and the calcined coupons is shown in Figures 5(a) and 5(b); significant morphological changes could be seen. Quantitative elemental analysis yielded a composition that corresponds to TiO_2_ within the permissible limits of errors. 

Hydrothermal processing has also been employed by others to create TiO_2_ coating on Ti plates. For example, Wang et al. [10] used hydrothermal treatment of Ti in 10 M NaOH solution in the temperature range of 140–200°C for time periods varying between 2 and 6 h. The autoclaved Ti substrates were soaked in 0.1 M HCl solution for 12 h followed by washing with DI water and annealing at 300–500°C for 2 h. This helped to create TiO_2_ nanoarrays.

Zuruzi and MacDonald [11] employed a process called “lift-off technique” to create titania nanoarrays on Ti surface, using aqueous H_2_O_2_ solution. The disadvantage of this procedure is the use of fluoroform, which is known to be a potent greenhouse gas in addition to being toxic. In comparison, the procedure employed in the current work is quite mild where either hydrogen peroxide solution or 5 M NaOH was used for short duration and at relatively lower temperatures.

### 3.3. Anodized Ti Substrates

**Figure 6:**
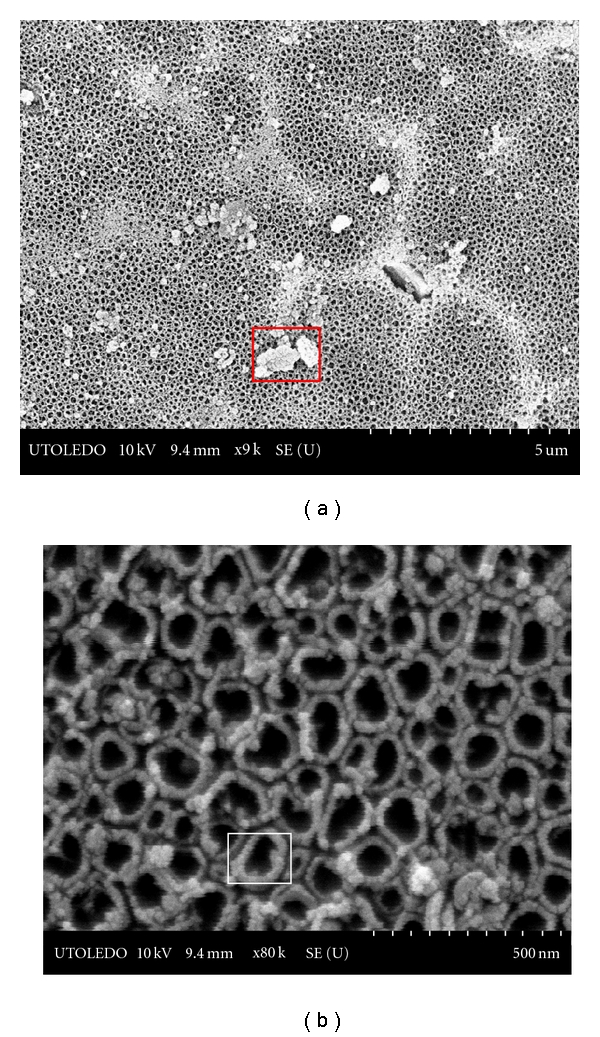
SEM images of TiO_2_ nanotubes created on cp Ti plate anodized for 1 h.

**Figure 7:**
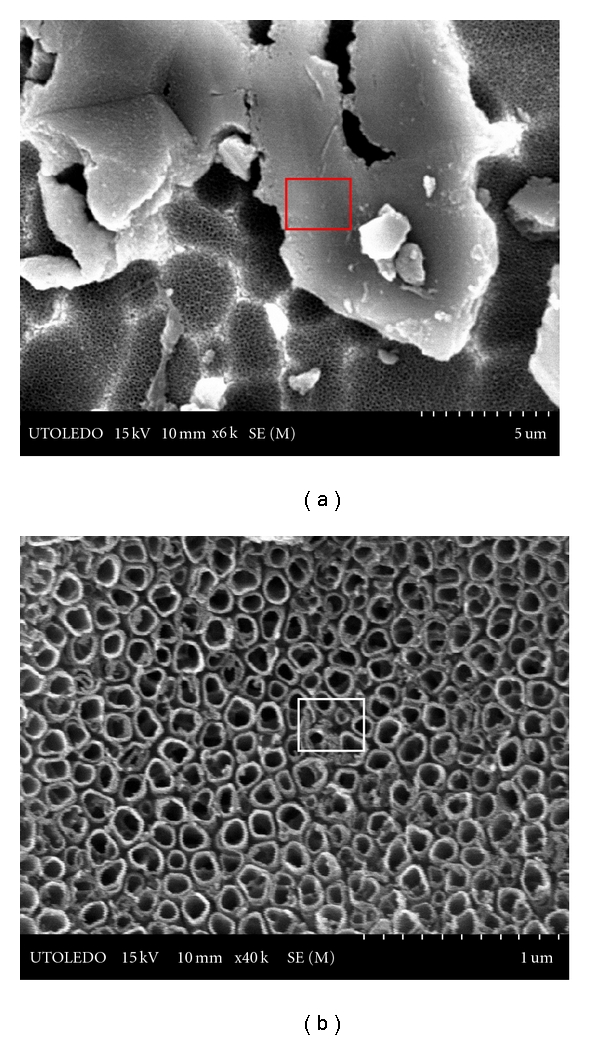
SEM images of TiO_2_ nanotubes created on cp Ti wire anodized for 1 h.

Creation of uniformly distributed nanostructured titania films on Ti plates and wires via anodization in 0.5 M H_3_PO_4_ + 0.14 M HF mixture at a voltage of 21 V is evidenced from the SEM images shown in Figure 6 for Ti plate and Figure 7 for wire that were anodized for 1 h. 

Elemental analyses on different locations of the specimen anodized for 1 h showed the presence of aluminum in addition to titanium and oxygen. This is due to the fact that commercially pure Ti contains small amount of aluminum; upon anodization, aluminum concomitantly migrates to the surface and also gets oxidized to Al_2_O_3_. This is corroborated by the data shown in Table 2.

However, as can be seen, the distribution of aluminum is far more widespread in Ti plates (from Titanium Inc.), compared to wire; evidently, in the case of plates, pure TiO_2_ nanotubes were not generated. We ascribe this difference to the availability of larger surface area in the case of plates that facilitated the migration of aluminum to be more universal than localized. This could be seen by the presence of aluminum as small intertwined rings on top of individual TiO_2_ nanotubes formed on plates, in Figure 6. 

**Figure 8:**
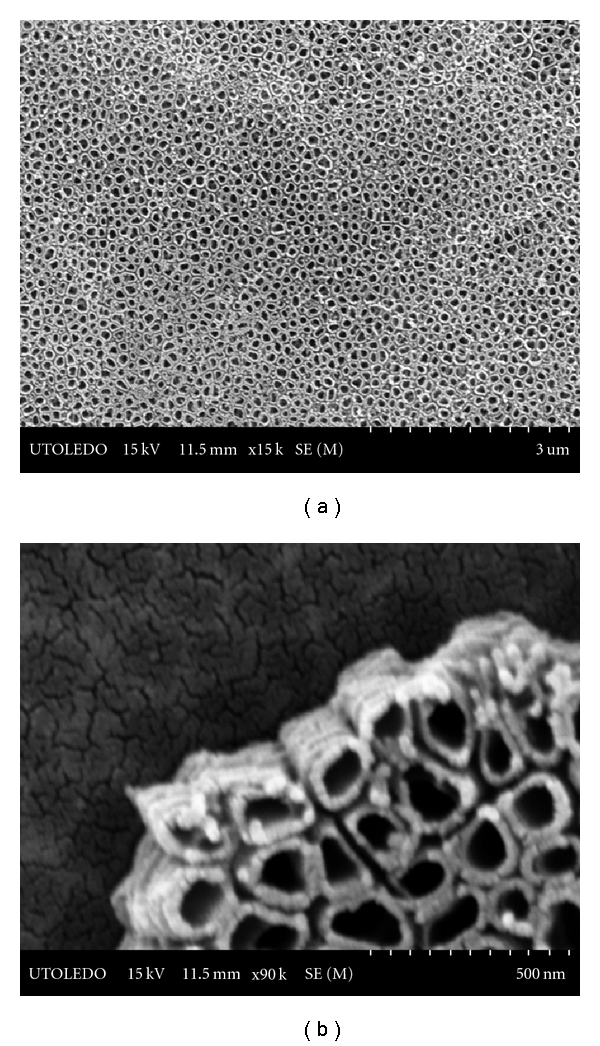
SEM images of the Ti plate anodized for 2 h.

Interestingly, the Ti plates anodized for 2 h under identical experimental conditions, showed the development of a consistently uniform nanotubular structure, as can be seen from Figure 8; the nanotubes are about 100–150 nm in diameter and ~300 nm in height. 

Also, from the EDS analysis results collected at random spots on the anodized plate, no evidence of aluminum was found on the film. It is likely that longer anodization leads to the dissolution of aluminum and/or aluminum oxide. This notion was strengthened by the results on anodization experiments carried out for even longer period.

In the case of Ti plates anodized for 4 h, it was found that the characteristic nanotubular features were totally destroyed. Moreover, in addition to the drastic variation in the morphological features, any titania film, if formed, also was destroyed; EDS analysis confirmed the presence of elemental Ti alone and no aluminum was detected.

This clearly shows that the optimum duration for Ti plate anodization is 2 h and, therefore, among all the plates, those anodized for 2 h were used in the bactericidal testing against *E. coli*. 

Mor et al. [12] have reported creating TiO_2_ nanotubes by anodization using Ti foil as the anode and Pt as cathode, in a mixture of 2.5 wt% HNO_3_ and 1 wt% HF aqueous solution for 1 to 4 h at temperatures ranging between 5 and 50°C; the anodization voltage of 20 V and 50°C was reported to yield the best results. Mohapatra et al. [3] reported creating highly ordered nanotubes of TiO_2_ by anodization of Ti foils (0.2 mm thick) in a mixture of 0.5 M H_3_PO_4_ and 0.14 M NaF at room temperature and application of 20 V. In comparison, our modified method was successful in creating nanotubes on 2 mm thick Ti plates at room temperature.

### 3.4. Evaluation of Bactericidal Efficacy

A detailed systematic evaluation protocol was drawn and carried out in order to unequivocally establish that the bacterial necrosis was most effective in the presence of titania films on cp Ti plates and wires, and that both irradiation and photocatalyst were needed.

Recently, we demonstrated the bactericidal efficacy of pure and Fe-doped titania nanofibers made by electrospinning [1] towards *E. coli* upon activation by UV (*λ* = 365 nm), multiphoton IR laser (*λ* = 820 nm) and IR (*λ* = 830 nm). It was also found that the illumination of *E. coli* broth by these radiations alone did not cause bacterial deactivation and necrosis [13]. It should be pointed out that unlike the free-standing fibers which are interconnected by particles whose average size is 30–40 nm, cp Ti and implant coupons used in this work were about 2 mm thick; even the Ti wire was 1.024 mm in thickness. Thus, it was imperative that in order to obtain appreciable bacterial mitigation, duration of photoactivation should be somewhat longer than that used in the case of nanofibers [1].

**Figure 9:**
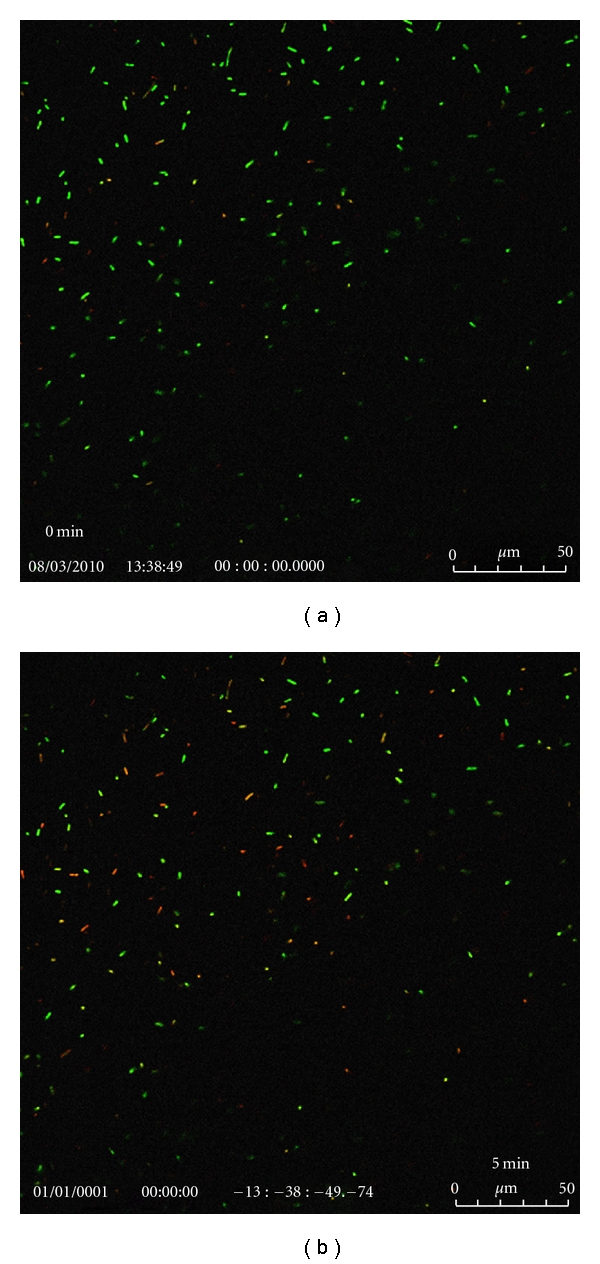
Confocal images of the bacterial colonies at different times in suspension containing Henkel Ti plate (autoclaved in 5 M NaOH at 100°C/2 h) and exposed to a handheld IR laser (*λ* = 808 nm) for 30 s.

**Figure 10:**
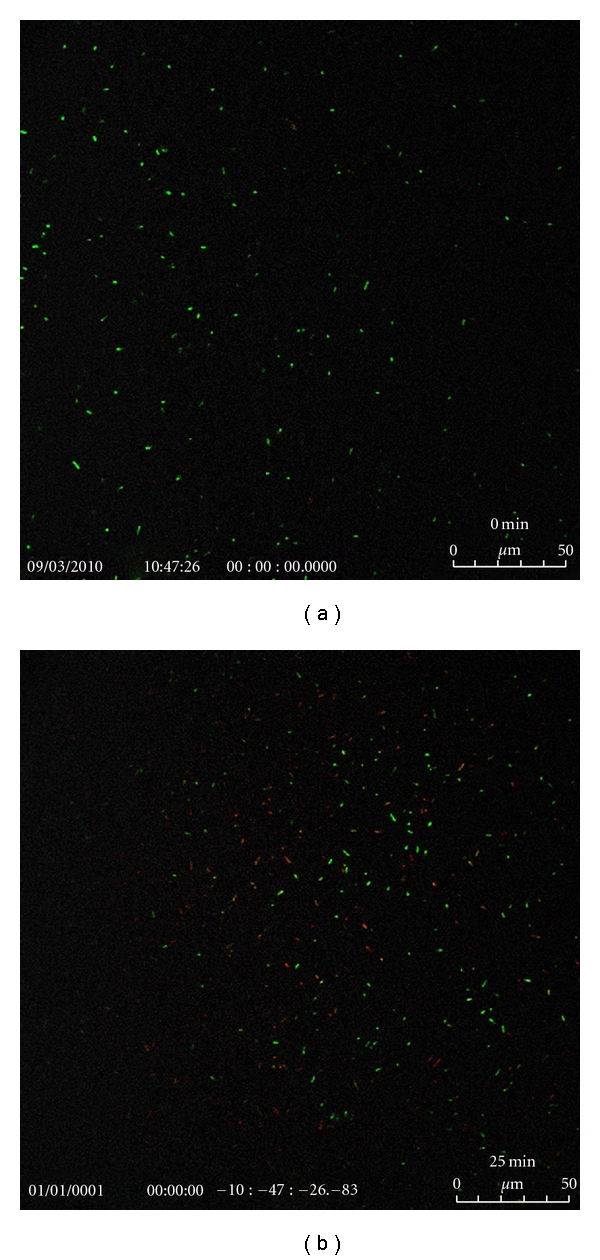
Confocal images of the bacterial colonies at different times in suspension containing Ti plate (Titanium Inc.; anodized for 2 h and chemically etched in EtOH/HF mixture for 5 s) and exposed to a handheld IR laser (*λ* = 808 nm) for 24 s.

**Figure 11:**
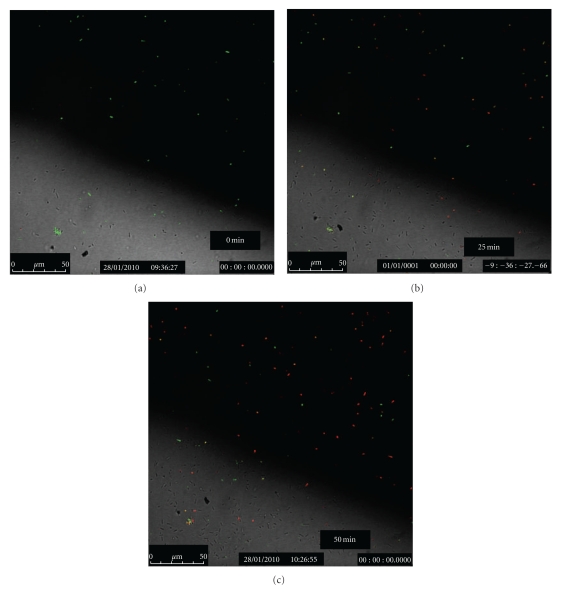
Confocal images of the bacterial colonies at different times in suspension containing Ti wire anodized for 1 h and exposed to a handheld IR laser (*λ* = 808 nm) for 12 s.

The confocal images of bacterial colonies after exposing the autoclaved or anodized Ti plates and wires to photoactivation by a handheld IR laser (*λ* = 808 nm) for various durations are shown in Figures 9, 10, and 11. Green pixels represent live cells while red pixels are for the dead cells. 

**Figure 12:**
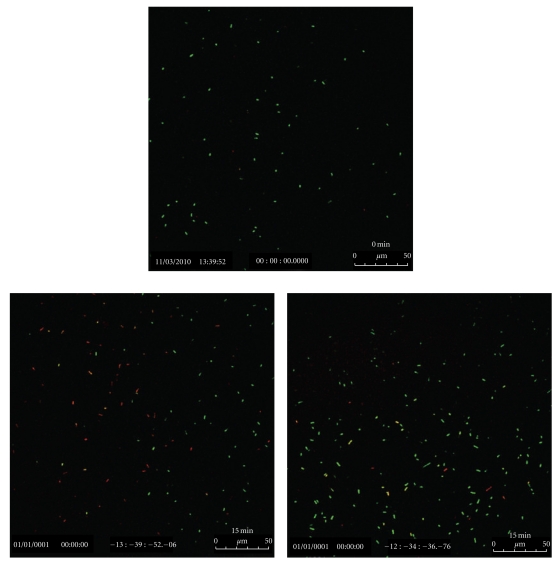
Confocal images of the bacterial colonies after 15 min in suspension containing: (right side) Henkel Ti plate autoclaved in 5 M NaOH at 100°C/2 h and (left side) Ti plate (Titanium Inc.; anodized for 2 h and chemically etched for 5 s in EtOH/HF) exposed to UV beam for 20 min.

The confocal data shown above, led to the conclusion that, among all Ti samples tested with the IR laser, two sets of results were most promising: (i) the Henkel Ti plates autoclaved in 5 M NaOH at 100°C for 2 h, and (ii) the plates from Titanium Inc. that were anodized for 2 h and chemically etched for 5 s in EtOH/HF mixture. In order to make a realistic comparison of the data collected on IR-activated specimen, parallel experiments were also carried out with UV light exposure on the two plates that worked well with IR beams. The autoclaved Henkel plates were found to be more effective than those anodized for 2 h. These results are shown in Figure 12.

The assessment of bactericidal effects of TiO_2_ particle irradiated by UV light has been examined by researchers to control bacterial infections in clinical applications. Koseki et al. [14], for example, used a suspension of *Staphylococcus aureus* of concentration 1 × 10^5^ cfu/mL (colony-forming units) in a solution containing 19 *μ*g/mL of TiO_2_ particles and irradiated them for 1 h with UV light (1.82 mW/cm^2^) in one case and with fluorescent light (80 *μ*W/cm^2^) in the other. It was found that the bacterial survival rate decreased steadily, reaching 9.4% after exposure to UV and 10.9% after exposure to fluorescent light.

Yu et al. [15] have reported the fabrication of Fe-doped TiO_2_ films on stainless steel substrates by dip coating followed by calcination and their use as antibacterial agents for sterilization against *Bacillus pumilus*. In this case, a suspension of *Bacillus pumilus* of concentration 1 × 10^7^ cfu/mL (colony-forming units) was placed onto the TiO_2_-coated stainless steel plate which was irradiated by a UV lamp (intensity rating 630 *μ*W/cm^2^; *λ* = 365 nm). Their results showed that the active *Bacillus pumilus* colonies on the titania films subjected to UV illumination decreased by 50% after 2 h of exposure.

In a recent work, Oka et al. [5] studied the inhibition of bacterial colonization of methicillin-resistant *Staphylococcus aureus* (MRSA) suspensions (1 × 10^8^ cfu/mL) on TiO_2_ film prepared by direct oxidization of pure titanium substrate. In this case, the titania coating on Ti was created by etching the latter with 5–10% HF solution followed by soaking in aqueous H_2_O_2_ for 2 days. The MRSA suspension on the implant was exposed to the ultraviolet A (UVA) light for 60 min and the number of colonizing bacteria was estimated. The bactericidal ability of the photocatalyst became apparent after 60 min, when the bacteria had almost disappeared; only about 7% bacteria were found alive. The number of colonizing bacteria on photocatalytic pins also decreased significantly *in vivo* as well. The titania film was found to be quite effective even against resistant bacterial colonization.

Table 3 summarizes the results of the present work and compares them with those reported on bactericidal efficacy of titania nanofibers made via electrospinning [1] and titania powder, using UV radiation and other types of microorganisms. As is well known, the UV radiation has higher photon energy than its IR counterpart. However, calculations based on the intensity of the incident beam and the time of exposure in each case, show that the number of photons incident per unit area during the exposure time is higher in the case of the present work using IR beam. This, in turn, translates into higher efficiency of the IR light compared to UV by several orders of magnitude (photons/cm^2^), since there are more photons for the time duration in comparison to the UV; this explains why the IR method used in this work, even for the shortest period of exposure, was much more effective than longer exposure by UV in the cases reported in the literature. We believe that the demonstration of the efficacy of IR light towards infection mitigation in terms of biocidal activity of titania is the first of its kind.

From the foregoing discussion, it is clear that a judicious combination of catalytic artifacts of titania and incident photons is quite effective in inhibiting bacterial colonization. While ultraviolet beam is effective in treating bacterial infection, it however, necessitates longer exposure to be of quantitative value; in some cases, the exposure could last from 20 to 60 min. In comparison, the present work demonstrates that better bactericidal activities were observed with IR exposure for a far shorter duration. Furthermore, combination of novel and benign methodologies of creating titania films and nanotubular configurations with infrared excitation in mitigating colonization is reported for the first time. This opens up the possibility of using an orthopedic implant with photoactive coating irradiated by a very short pulse of an infrared beam, as an infection mitigation device.

It should be pointed out that while *γ*-radiations have been used in other processes, the authors are not aware of any with regard to their use in conjunction with titania. Moreover, the gamma rays are a form of ionizing radiation and as such, they pose health hazard. They, like neutrons, are more penetrating, causing diffusive damage to the body tissues (e.g., radiation sickness, increased probability of cancer). External radiation exposure should also be distinguished from internal exposure, due to ingested or inhaled radioactive substances, which, depending on the substance's chemical nature, can produce both diffuse and localized internal damage.

Consequently, gamma rays would kill the bacteria but also harm the human host from the inside out. On the other hand, the use of an alternative sterilization agent, namely, ethylene oxide (EtO) is not benign: its shortcoming is that it may leave toxic residuals that would cause adverse reactions after implantation. Thus, its efficacy towards bacterial resistance or necrosis is questionable. On the other hand, the energetic photons generated on the photoactivated implants, lead to the generation of free radicals that oxidize and kill the bacteria more efficiently.

This is the first feasibility study on the use of IR radiation on its bactericidal potential. Though the successful implementation of the adopted strategy has been demonstrated, this by no means is an indication that the positive, but nevertheless mediocre, level of necrosis (~60–70% reduction) is acceptable as a final outcome. We recognize and emphasize that more work needs to be done to attain clinically meaningful levels of reduction which requires orders of magnitude reduction in viability. These practical goals could be achieved by optimizing the proposed method by, for instance, somewhat longer IR radiation exposure using a radiation source of higher strength (power), and so forth.

With regard to the next steps in terms of evaluating the titania-coated implants, we plan to gauge the effectiveness (efficacy and/or toxicity) of the material on the underlying wounds or infections. Experiments are in progress to examine the efficacy of titania against *S. aureus*, since it is a BSL2 gram-positive bacterium while *E. coli* is a gram-negative BSL1 agent. The gram-positive bacterium has stronger membrane and is likely to be more resistant to the reactive free radicals created with the photoexcitation of titania. At this juncture, it is worth pointing out that it is likely that the TiO_2_ micro/nanocoatings could lead to discontinuities with the potential to reduce the fatigue strength of the devices coated with TiO_2_. We will attempt to address these issues, by doing coated-implant evaluations as per the ASTM standards, in subsequent studies.

## 4. Conclusions

Nanostructured titania coatings were created by mild and benign techniques of hydrothermal processing and anodization on titanium substrates. The films were characterized thoroughly for their structural integrity and microstructural features. Their efficacy for the inhibition of bacterial colonization of *E. coli* was evaluated by irradiating with a handheld IR laser and UV beams for durations between 12 s and 30 min. The confocal microscopic results demonstrated that IR exposure for short duration was quite effective in bactericidal activities.

To expand the application of titania coatings on implants for wound disinfection, assessing their effect on the bacterial spores, commonly accepted by the medical device community, such as *Bacillus pumilus* and *Staphylococcus aureus* is under progress.

## Figures and Tables

**Table 1:** Semiquantitative compositional analyses of the films formed on Ti coupons autoclaved for 1/2 and 1 h.

Element	Autoclaved at 80°C for			
	1/2 h		1 h	
	wt.%	at.%	wt.%	at.%
O	72.77	88.89	35.01	61.73
Ti	27.23	11.11	64.99	38.27

Total	100	100	100	100

**Table 2:** Compositional analyses at different location on the Ti plate and wire anodized for 1 h.

Element	Anodized Ti plate				Anodized Ti wire			
	Red zone		White zone		Red zone		White zone	
	wt.%	at.%	wt.%	at.%	wt.%	at.%	wt.%	at.%
O	16.32	29.4	21.29	34.21	37.58	62.28	26.5	51.91
Ti	40.28	24.24	22.18	11.9	55.05	30.48	73.5	48.09
Al	43.39	46.36	56.54	53.88	7.37	7.24	0	0

Total	100	100	100	100	100	100	100	100

**Table 3:** Summary of the results and comparison with the literature.

Bacteria	Light source	*λ* (nm)	Exposure time	Intensity (W/cm^2^)	Photon energy (J)	Photon/area^‡^	Survival rate (%)	Reference
*Staphylococcus aureus*	UV	352	60 min	1.82 × 10^−3^	5.647 × 10^−19^	1.16 × 10^19^	9.4	Koseki et al.^#^ [14]
*Bacillus pumilus*	UV	365	120 min	630 × 10^−6^	5.446 × 10^−19^	8.33 × 10^18^	≤50	Yu et al.^#^ [15]
*Methicillin-resistant Staphylococcus aureus* (MRSA)	UVA	365	60 min	1.1 × 10^−3^	5.446 × 10^−19^	7.27 × 10^18^	7	Oka et al.^#^ [5]
*Escherichia coli*	IR/MP	820	3 s	2 × 10^6^	2.424 × 10^−19^	2.48 × 10^25^	10	Azad et al.^$^ [1]
*Escherichia coli*	IR/laser	808	30 s	5	2.460 × 10^−19^	6.10 × 10^20^	60	This work^$$^
*Escherichia coli*	UV	365	20 min	350 × 10^−6^	5.466 × 10^−19^	7.68 × 10^17^	60	This work^$$^

^‡^Incident photons per unit area during exposure time are obtained by multiplying the ratio of intensity (W/cm^2^) and photon energy (J) with the time of exposure (s).

^#^Titania powder.

^$^Titania nanofibers.

^$$^Titania coating on cp Ti.
